# Adolescents' leisure activities, parental monitoring and cigarette smoking - a cross-sectional study

**DOI:** 10.1186/1747-597X-6-12

**Published:** 2011-06-06

**Authors:** Hui Guo, Anthony I Reeder, Rob McGee, Helen Darling

**Affiliations:** 1 Cancer Society of New Zealand Social and Behavioral Research Unit (SBRU), Department of Preventive and Social Medicine, Dunedin School of Medicine, University of Otago, Dunedin, New Zealand; 2 Oritain Global Ltd., 8 Pacific St., Dunedin, 9010, New Zealand

## Abstract

**Background:**

Adolescent participation in leisure activities is developmentally beneficial, but certain activities may increase health compromising behaviours, such as tobacco smoking. A limited range of leisure activities has been studied, with little research on out-of-school settings where parental supervision is a potential protective factor. Tobacco smoking is an important, potentially modifiable health determinant, so understanding associations between adolescent leisure activities, parental monitoring, demographic factors and daily smoking may inform preventive strategies. These associations are reported for a New Zealand adolescent sample.

**Methods:**

Randomly selected schools (*n *= 145) participated in the 2006 Youth In-depth Survey, a national, biennial study of Year 10 students (predominantly 14-15 years). School classes were randomly selected and students completed a self-report questionnaire in class time. Adjustment for clustering at the school level was included in all analyses. Since parental monitoring and demographic variables potentially confound relations between adolescent leisure activities and smoking, variables were screened before multivariable modelling. Given prior indications of demographic differences, gender and ethnic specific regression models were built.

**Results and Discussion:**

Overall, 8.5% of the 3,161 students were daily smokers, including more females (10.5%) than males (6.5%). In gender and ethnic specific multivariate analysis of associations with daily smoking (adjusted for age, school socioeconomic decile rating, leisure activities and ethnicity or gender, respectively), parental monitoring exhibited a consistently protective, dose response effect, although less strongly among Māori. Attending a place of worship and going to the movies were protective for non-Māori, as was watching sports, whereas playing team sport was protective for all, except males. Attending a skate park was a risk factor for females and Māori which demonstrated a strong dose response effect.

**Conclusions:**

There were significant differences in the risk of daily smoking across leisure activities by gender and ethnicity. This reinforces the need to be alert for, and respond to, gender and ethnic differences in the pattern of risk and protective factors. However, given the consistently protective, dose response effect of parental monitoring, our findings confirm that assisting oversight of adolescent leisure activities may be a key component in public health policy and prevention programmes.

## Background

It has long been known that involvement in leisure activities can assist adolescents in exerting personal control over their environments and developing a positive sense of identity through their actions [1]. Physical activities programmes can 'help children develop social skills, improve mental health, and reduce risk-taking behaviours.'[2] Participation in organised activities, such as team sport, is often associated with reduced involvement in antisocial behaviours and substance use,[3-5] including tobacco smoking [6]. In particular, team sports involvement has been associated with lower levels of cigarette smoking,[7] and consistent, high and multiple participation in team sports over a number of years may provide particularly effective protection when compared with more intermittent patterns of participation [8].

A possible explanation for these observations is that organised activities may be protective by facilitating pro-social group membership [3,9]. The protective effect may be attributable to displacement, whereby the time available to spend in unstructured activities with antisocial peers is reduced [6]. A New Zealand study found that moderate or high levels of involvement in physical activity, but not team sports at age 15 years, were associated with antisocial behaviours for both sexes at age 18 years [10]. Using structural equation modelling, a recent longitudinal study found significant associations between adolescents' activities at baseline and tobacco smoking 24 months later [11]. Interestingly, the pathways differed by activity type and sex. For girls, an indirect path from baseline participation in school clubs and activities lay through reduced association with 'problem peers' at 15 months follow up, whereas for boys baseline participation in team sports was linked to tobacco smoking through on-going team sports participation at 15 months. Overall, it is possible that less structured leisure activities may leave participants at increased risk of experimentation with health compromising behaviours such as tobacco smoking, whereas other activities and settings, such as team sports and club membership, may provide a more protective social and physical environment [6].

Faith-based activities have also been identified as potentially protective against substance use and antisocial behaviours [3]. When adjusted for baseline smoking, religious activity was associated with tobacco smoking at 24 months for both sexes [11]. For girls, baseline religious activity indirectly reduced tobacco smoking at 24 months by reducing exposure to problem peers at 15 months, whereas for boys the path lay through religious activity at 15 months, which was directly related to lower levels of tobacco smoking at 24 months. These differences suggest the potential informative value for policy development of gender specific analyses, and the same may be true for analysis by ethnicity [12].

To date, the range of leisure activities studied in relation to tobacco use has been limited, with relatively little research on out-of-school settings, but there is evidence of a protective effect for some other problem behaviours, such as marijuana use [13]. In the study reported here, we examined daily cigarette smoking in a variety of social contexts including attending a place of worship, which suggests a shared set of values or sense of community; involvement in team sports and voluntary work, which may promote social and physical well-being; and engagement in musical activities, movies and skate boarding, which represent contexts perhaps most likely to be influenced by informal group processes.

Parents may have different degrees of awareness of the activities in which their adolescents participate, but parental monitoring is a potentially important protective factor against adolescent smoking [14]. For example, secondary school students who were home alone on two or more days per week were more likely to smoke than those having parental supervision five or more times a week [15]. Of particular relevance in the present context, parental monitoring may also influence an adolescent's choice of activities and potentially confound the observed relationships between leisure activities and adolescent smoking. Accordingly, in this study we used multivariable modelling to examine whether any observed effects associated with leisure activities may simply reflect less parental monitoring or whether parental monitoring and various leisure activities are each independently associated with tobacco smoking.

Given that tobacco use is an important and potentially modifiable determinant of health, understanding more about the associations of leisure activities, socio-demographic factors and parental monitoring with adolescent smoking may help inform and guide the development and targeting of preventive policies and protective strategies. Most adult smokers started smoking in adolescence, so identifying which factors may increase risk or exert protective effects against smoking during that period of life may be particularly important for the design and targeting of preventive interventions [11].

## Method

### Sample selection

The Year 10 Youth In-depth Survey (YIS) is a biennial survey carried out by the Health Sponsorship Council of NZ with methods and key measures from the international Global Youth Tobacco Survey (GYTS) [16]. Our study uses data from the 2006 survey of Year 10 (predominantly 14-15 year-old) students from randomly-selected secondary schools. The survey used a self-report questionnaire administered during class time. Of the 186 randomly selected schools, 145 or 78% agreed to participate [17]. Ethical approval for analyzing the data was obtained through the Department of Preventive and Social Medicine, following University of Otago procedures.

### Measures

The outcome variable, daily smoking, was assessed by the question "how often do you smoke now?" with those students who responded "at least once a day" categorized as daily smokers. Students were also asked the frequency of engaging in a variety of activities during the month preceding the survey, including: attending a place of worship, attending a music event or concert, visiting a music shop, watching a movie in a theatre, visiting a skate-park, playing sports for a team during the weekend or after school, going somewhere to watch a sports game or event, and doing community voluntary work. The response categories for the past month were: 0, 1-3, or 4+ times (i.e., at least weekly). Parental monitoring outside of school hours was assessed using a 4-item scale adapted from the NZ Youth 2000 survey[18] and the Centers for Disease Control (CDC) "Got a minute" parenting campaign measurement tool [19]. Participants were asked whether they agreed or disagreed with the following statements, "My parents or caregivers": "generally know what I spend my pocket money on"; "have rules about when I can go out with my friends"; "often have no idea where I am, when I am away from my home"; and "If I break any important rules that my parents or caregivers have set I always get into trouble." The scale demonstrated acceptable internal consistency (Cronbach's alpha = 0.69). It was not considered appropriate to ask students about household income in order to estimate family socioeconomic status (SES). Instead, school socio-economic decile, which provides a measure of the relative poverty of the parents or care-givers of students at a school, was used as a proxy measure of SES. Deciles 1 and 10, respectively, include the 10% of schools drawing students from the lowest and highest socioeconomic communities. For the purpose of descriptive comparison the scale was collapsed into three categories with deciles 1 to 4 being 'low', 5 to 7 'mid' and 8 to 10 'high' SES, [16] but treated as a continuous variable in the regression analyses.

### Statistical analyses

Stata version 10.1 was used for all the analyses [20]. As data were collected from individuals within selected schools, adjustment for clustering at the school level was included in all analyses. Weights were calculated from the total number of students in each school year for each randomly selected school. Simple logistic regression was used to examine the associations between potential predictor variables and the binary outcome variable adolescent daily smoking. As the bivariate modeling was exploratory, a *p*-value of ≤0.20 was used as the cut off value for the selection of explanatory variables for multivariate analysis [21]. Four multiple logistic regression models (for males and females, Māori and non-Māori) were used to examine the associations of parental monitoring scores and participation in the selected activities with the binary outcome variable, adolescent smoking. These four models were adjusted for student age, school socioeconomic decile and either ethnicity or sex, as appropriate. The results are presented as unadjusted or adjusted odds ratios (OR) with 95% confidence intervals (CI's). The relation between each pair of the predictor variables was checked using Spearman's correlations so that if any variables were strongly correlated (|r| < 0.70), only a single variable would be included in each model to minimize problems associated with collinearity. However, the highest correlation was between watching a sports event and playing sport (r = 0.44).

## Results

Students (*n *= 39) who did not provide full data about their age, sex, ethnicity and tobacco smoking status were excluded from the study, leaving 3,161 participants: 51% females and 49% males. Of these, 8.5% and more females (10.5%) than males (6.5%) were daily smokers. More Māori students (20.5%) were daily smokers than non-Māori (7.2%). The socio-demographic characteristics of the sample are presented in Table 1.

**Table 1:** Socio-demographic characteristics of the YIS 2006 sample

	Current daily smokers		YIS 2006 sample	
	(n = 269)		(n = 3161)	
	n	%*	n	%*
**Age**				
13 yrs	2	0.7	24	0.8
14 yrs	166	61.7	2035	64.3
15 yrs	96	35.7	1069	33.8
16 yrs	4	1.5	24	0.8
17 yrs	1	0.4	5	0.2
≥ 18 yrs	0	0	4	0.1
**Sex**				
Female	168	62.5	1606	50.8
Male	101	37.5	1555	49.2
**Ethnicity**				
NZ European	90	33.5	1724	54.6
Māori	134	49.8	654	20.7
Pacific Island	25	9.3	271	8.6
Asian	3	1.1	294	9.3
Other	17	6.3	218	6.8
**School decile **				
low	114	34.5	849	26.9
mid	108	41.4	1198	37.9
high	47	24.1	1114	35.2

*Weighted % (probability weights assigned at individual student level)

The distribution of all students and daily smokers engaging in the eight leisure activities are presented in Table 2 along with the distribution of parental monitoring scores.

**Table 2:** Descriptive results: parental monitoring scores and participation in activities

	Current daily smokers		YLS 2006 sample	
	N	%*	n	%*
**Parental monitoring scores**				
0	25	10.6	219	7.1
1	61	23.7	375	12.1
2	72	27.0	696	22.5
3	72	31.1	931	30.2
4	27	7.6	867	28.1
**Gone to a place of worship**				
Not in past month	97	77.4	1865	65.0
1-3 times	110	8.7	453	15.6
4 or more times	40	14.0	565	19.4
**Watched sports game or event**				
Not in past month	68	35.5	1036	34.9
1-3 times	97	38.0	1165	39.0
4 or more times	73	26.5	774	26.0
**Played team sports **				
Not in past month	130	57.1	1180	40.5
1-3 times	33	14.1	489	16.5
4 or more times	75	28.8	1290	43.0
**Done voluntary work **				
Not in past month	196	88.0	2480	90.2
1-3 times	7	3.7	163	5.9
4 or more times	18	8.3	108	3.9
**Gone to the movies**				
Not in past month	97	40.3	1249	41.7
1-3 times	110	42.8	1508	50.2
4 or more times	40	16.9	232	8.1
**Gone to a skate park**				
Not in past month	118	54.0	2152	73.9
1-3 times	64	25.6	524	18.3
4 or more times	54	20.4	221	7.8
**Gone to a music event/concert **				
Not in past month	147	60.1	2099	73.0
1-3 times	81	35.1	724	24.6
4 or more times	11	4.8	68	2.4
**Gone to a music shop**				
No visits	98	39.4	1439	49.5
1-3 times	97	41.5	1131	38.4
4 or more times	44	19.1	356	12.1

*Weighted % (probability weights assigned at individual student level)

The results of the logistic regression analyses by sex and by ethnicity are presented in Tables 3 and 4, respectively.

**Table 3:** Unadjusted and adjusted OR for male and female daily smoking and adolescent activities, YIS 2006

	Males				Females			
	
	Unadjusted	Overall	**Adjusted**^1^	Overall	Unadjusted	Overall	**Adjusted**^1^	Overall
	OR (95% CI)	p-value	OR (95% CI)	p-value	OR (95% CI)	p-value	OR (95% CI)	p-value
**Parental monitoring score**		< 0.001		0.003		< 0.001		< 0.001
0	1.0		1.0		1.0		1.0	
1	0.70 (0.60, 0.82)		0.77 (0.65, 0.92)		0.65 (0.57, 0.75)		0.73 (0.62, 0.86)	
2	0.50 (0.37, 0.67)		0.60 (0.42, 0.84)		0.43 (0.33, 0.56)		0.53 (0.38, 0.73)	
3	0.35 (0.22, 0.55)		0.46 (0.28, 0.77)		0.28 (0.19, 0.42)		0.38 (0.24, 0.63)	
4	0.25 (0.13, 0.46)		0.36 (0.18, 0.71)		0.18 (0.11, 0.31)		0.28 (0.15, 0.53)	
**Gone to a place of worship**		0.240		N/A		0.037		0.212
No visits	1.0				1.0		1.0	
1-3 visits	0.44 (0.17, 1.17)				0.65 (0.38, 1.10)		0.77 (0.43, 1.39)	
4 or more visits	1.02 (0.51, 2.03)				0.50 (0.27, 0.92)		0.55 (0.27, 1.13)	
**Watched sports game or event**		0.534		N/A		0.081		0.431
No visits	1.0				1.0		1.0	
1-3 visits	0.78 (0.44, 1.38)				1.86 (1.08, 3.21)		1.48 (0.82, 2.68)	
4 or more visits	1.12 (0.57, 2.19)				1.50 (0.86, 2.60)		1.30 (0.69, 2.44)	
**Played team sports **		0.068		0.096		< 0.001		0.002
No visits	1.0		1.0		1.0		1.0	
1-3 visits	0.54 (0.24, 1.20)		0.63 (0.26, 1.55)		0.64 (0.38, 1.16)		0.51 (0.27, 0.97)	
4 or more visits	0.52 (0.28, 0.96)		0.46 (0.23, 0.93)		0.42 (0.27, 0.63)		0.40 (0.24, 0.67)	
**Done voluntary work **		0.003		0.020		0.622		N/A
No visits	1.0		1.0		1.0			
1-3 visits	0.28 (0.04, 1.86)		0.22 (0.03, 1.92)		0.68 (0.27, 1.69)			
4 or more visits	4.02 (1.75, 9.24)		3.51 (1.26, 9.32)		1.23 (0.53, 2.82)			
**Gone to the movies**		< 0.001		0.260		0.004		0.184
No visits	1.0		1.0		1.0		1.0	
1-3 visits	0.99 (0.58, 1.68)		0.94 (0.54, 1.64)		0.84 (0.54, 1.31)		0.97 (0.62, 1.54)	
4 or more visits	3.72 (1.87, 7.40)		1.83 (0.80, 4.17)		2.39 (1.26, 4.54)		2.24 (0.91, 5.50)	
**Gone to a skate park**		< 0.001		0.027		< 0.001		< 0.001
No visits	1.0		1.0		1.0		1.0	
1-3 visits	1.52 (0.73, 3.17)		1.14 (0.54, 2.39)		2.96 (1.98, 4.43)		1.95 (1.21, 3.15)	
4 or more visits	4.24 (1.95, 7.90)		2.61 (1.28, 5.32)		9.45 (4.96, 18.01)		5.13 (2.49, 10.57)	
**Gone to a music event/concert **		0.001		0.092		0.010		0.135
No visits	1.0		1.0		1.0		1.0	
1-3 visits	2.46 (1.54, 3.91)		1.78 (1.10, 2.98)		1.17 (0.77, 1.78)		0.93 (0.58, 1.49)	
4 or more visits	2.11 (0.52, 8.58)		1.86 (0.13, 6.56)		3.41 (1.56, 7.45)		2.56 (0.97, 6.81)	
**Gone to a music shop**		0.089		0.520		0.062		0.949
No visits	1.0		1.0		1.0		1.0	
1-3 visits	1.21 (0.65, 2.24)		1.21 (0.63, 2.33)		1.09 (0.73, 1.65)		1.01 (0.63, 1.64)	
4 or more visits	2.40 (1.18, 4.90)		1.64 (0.70, 3.81)		1.81 (1.09, 3.02)		0.91 (0.46, 1.83)	

**Table 4:** Unadjusted and adjusted OR for Māori and non-Māori daily smoking by parental monitoring and activities

	Māori				Non-Māori			
	
	Unadjusted	Overall	**Adjusted**^1^	Overall	Unadjusted	Overall	**Adjusted**^1^	Overall
	OR (95% CI)	p-value	OR (95% CI)	p-value	OR (95% CI)	p-value	OR (95% CI)	p-value
**Parental monitoring scores**		0.008		0.030				< 0.001
0	1.0		1.0		1.0		1.0	
1	0.81 (0.70, 0.95)		0.82 (0.65, 0.92)		0.53 (0.33, 0.85)		0.51 (0.44, 0.60)	
2	0.66 (0.49, 0.90)		0.67 (0.47, 0.96)		0.28 (0.11, 0.73)		0.26 (0.20, 0.35)	
3	0.54 (0.34, 0.85)		0.55 (0.33, 0.94)		0.15 (0.04, 0.62)		0.14 (0.09, 0.21)	
4	0.44 (0.24, 0.81)		0.46 (0.22, 0.93)		0.08 (0.01, 0.53)		0.07 (0.04, 0.13)	
**Gone to a place of worship**		0.341		N/A		< 0.001		< 0.001
No visits	1.0				1.0		1.0	
1-3 visits	0.73 (0.37, 1.44)				0.11 (0.05, 0.22)		0.22 (0.08, 0.61)	
4 or more visits	0.65 (0.32, 1.31)				0.17 (0.09, 0.32)		0.30 (0.15, 0.60)	
**Watched sports game or event**		0.211		N/A		< 0.001		< 0.001
Not in past month	1.0				1.0		1.0	
1-3 times	1.57 (0.82, 3.02)				0.11 (0.08, 0.17)		0.43 (0.28, 0.64)	
4 or more times	1.03 (0.54, 1.98)				0.15 (0.10, 0.21)		0.77 (0.43, 1.38)	
**Played team sports **		< 0.001		< 0.001		< 0.001		< 0.001
Not in past month	1.0		1.0		1.0		1.0	
1-3 times	0.48 (0.26, 0.91)		0.41 (0.20, 0.87)		0.10 (0.05, 0.19)		0.33 (0.15, 0.73)	
4 or more times	0.44 (0.27, 0.70)		0.38 (0.23, 0.64)		0.08 (0.06, 0.12)		0.29 (0.16, 0.50)	
**Done voluntary work **		0.108		0.093		< 0.001		0.449
Not in past month	1.0		1.0		1.0		1.0	
1-3 times	0.38 (0.09, 1.63)		0.30 (0.06, 1.55)		0.17 (0.06, 0.46)		0.53 (0.13, 2.17)	
4 or more times	2.16 (0.83, 5.61)		2.26 (0.82, 6.25)		0.47 (0.22, 1.01)		1.82 (0.55, 6.02)	
**Gone to the movies**		0.131		0.310		< 0.001		< 0.001
Not in past month	1.0		1.0		1.0		1.0	
1-3 times	0.95 (0.57, 1.60)		1.15 (0.67, 1.97)		0.12 (0.09, 0.16)		0.46 (0.31, 0.66)	
4 or more times	1.89 (0.90, 3.97)		1.90 (0.83, 4.37)*		0.42 (0.22, 0.80)		0.89 (0.30, 2.62)	
**Gone to a skate park**		< 0.001		< 0.001		< 0.001		0.347
Not in past month	1.0		1.0		1.0		1.0	
1-3 times	1.73 (1.01, 2.96)		1.62 (0.72, 3.23)		0.42 (0.27, 0.64)		1.33 (0.73, 2.43)	
4 or more times	4.21 (2.25, 7.99)		4.74 (2.32, 9.69)		0.82 (0.47, 1.42)		1.81 (0.70, 4.70)	
**Gone to a music event/concert **		0.393		N/A		< 0.001		0.251
Not in past month	1.0				1.0		1.0§	
1-3 times	1.06 (0.61, 1.84)				0.38 (0.27, 0.54)		1.50 (0.92, 2.46)	
4 or more times	2.44 (0.67, 8.88)				0.72 (0.33, 1.57)		1.71 (0.35, 8.42)	
**Gone to a music shop**		0.150		0.053		< 0.001		0.142
No visits	1.0		1.0		1.0		1.0	
1-3 times	1.20 (0.72, 2.00)		1.03 (0.61, 1.77)		0.17 (0.12, 0.24)		0.58 (0.33, 0.99)	
4 or more times	1.77 (1.00, 3.16)		0.99 (0.46, 2.15)		0.32 (0.20, 0.51)		0.79 (0.40, 1.59)	

Parental monitoring exhibited a protective, dose response effect for both sexes, whereby increasing monitoring scores were associated with decreasing odds of daily smoking. Playing team sports demonstrated a protective effect, but only among females. In contrast, going to a skate park was positively associated with daily smoking, most strongly among females in the multivariable model. Having done voluntary work was a risk factor among males, but this was relatively weak in a multivariable context whereas going to the movies and a music event/concert lost significance as a risk factor for both sexes in the multivariable modeling.

Parental monitoring score was protective against daily smoking for non-Māori, exhibiting a strong dose response effect. However, for Māori, it became only weakly protective in a multivariable context. For non-Māori, going to a place of worship, watching sports, playing team sports and going to the movies were all protective and there were no statistically significant risk factors in the multivariable model. However, among Māori, playing team sports was protective and parental monitoring score was only weakly protective, whereas going to a skate park was a risk factor with strong dose response characteristics.

## Discussion

Adolescence is a pivotal period for psychosocial and physical development during which life experiences and social contexts can shape the positive-to-negative balance of outcomes [22]. Although quite a lot is known about family and peer effects on adolescent tobacco smoking, fewer studies have examined the possible influence of leisure activities and none, so far as we could ascertain, have done this in the context of multivariate analyses that also included parental monitoring as a potential predictor.

Our finding that parental monitoring was universally strongly protective against adolescent daily tobacco smoking is consistent with other recent evidence [12,23]. However, the effects observed those studies did not take into account the associations between tobacco smoking and participation in leisure activities. We also found evidence of a protective effect against smoking among those who engaged in extracurricular team sports, consistent with earlier US studies of adolescents who participated in school-based team sports [24-26]. However, we found that this only held true for females, which is the reverse of the relationship found in a recent longitudinal study [11]. It is possible that this may relate to cultural differences between the US and NZ regarding sports participation, and is worthy of further investigation. There is evidence that team sports participation can be a risk factor for other adolescent health risk behaviours, such as alcohol use, [9] but legislative control of smoking in shared indoor environments in NZ, including sports clubs, would tend to limit the risk of tobacco smoking.

We found some evidence of an increased risk of daily smoking among those who reported going to musical events, the movies and music shops, but in multivariate analysis only going to the movies was associated with daily smoking and as a protective factor among non-Māori. Going to a skate park was the strongest predictor, but in a multivariable context it was a statistically significant risk factor only for females and Māori, demonstrating a dose response effect in each case. Although clearly not universal in our sample, this finding is consistent with the UK research which suggests that street-oriented leisure activities are associated with a greater risk of tobacco smoking [27].

Although our data come from a national survey of Year 10 students with a reasonable participation rate (65.3%),[17] the most obvious limitation is the use of a cross-sectional design to test for associations of potential risk and protective factors with daily smoking [11]. In spite of strong associations between some of the variables, and evidence of dose-response relationships, there is insufficient evidence to assert causal relations. Further studies should include prospective assessment in order to better understand the temporal relationship between these factors and adolescent smoking.

## Conclusions

Study findings reinforce Kaufman and Fieden's statement that 'Young people should be studied within the broad social and environmental contexts in which they live' (p. S11) [28]. Better understanding of different aspects of youth leisure activities, and of parental monitoring, should help inform and strengthen the study of youth health behaviors and tobacco smoking prevention efforts. Our study findings reinforce the need to be alert for, and respond to gender[11] and ethnic[12] differences in the patterns of risk and protective factors. However, given our finding that parental monitoring score was a protective factor across gender and ethnicity, and demonstrated a consistent dose response effect, interventions that involve working with parents, such as the CDC's 'Got a minute' program,[19] are likely to be an important component for preventing adolescent tobacco smoking. That a less strong effect was observed for Māori adolescents may be related to high smoking rates among adult Māori, the reduction of which is currently a critical target for improving Māori Health, overall.

Greene and Bannerjee (2009) concluded that unsupervised time with adolescent peers was associated indirectly with smoking behavior through the mediation of association with delinquent peers [29]. This finding led those authors to suggest that "interventions designed to motivate adolescents without adult supervision to associate more with friends who engage in pro-social activities may eventually reduce adolescent smoking." However, this presents a challenge, especially given evidence that physical activity may be waning in many countries, particularly in clearly defined contexts such as school physical education, and organised sports [30]. This may also be true for NZ, but it is not clear because insufficient monitoring has been done. Given our finding of a protective effect for playing team sports, at least among females, such monitoring should be undertaken to help inform and guide policy and program development. In addition to the collection of more comprehensive data on a range of activities in successive cross-sectional waves, an important contribution would be made by the initiation of longitudinal studies to disentangle causality,[29] since the years of adolescence present policy opportunities with the potential to influence long term health outcomes. Any policy initiatives to increase participation in leisure activities should aim to promote those activities which optimize health gains while minimizing health risks, and include the monitoring of venues, such as skate parks. As noted earlier, there is also a need to pay attention to potential group preferences, taking into account gender and ethnic differences.

Tobacco smoking is a key behavioral outcome for health in the short term but, even more importantly, because those adolescents who smoke, in particular regular smokers, are likely to continue to smoke into adulthood. As stated elsewhere, 'once dependence is established, the majority of smokers will then continue to smoke for nearly 40 years. Experimentation with cigarettes in adolescence clearly has major long-term implications for individual and public health.'(p. 122)[31] Given the range of serious negative health outcomes linked with smoking, further study of risk and protective factors for tobacco smoking in adolescence has great potential public health and policy significance.

## Competing interests

Dr Reeder serves on the National Health Promotion Advisory Committee of the Cancer Society of New Zealand Inc. Prof McGee serves on the Board of the Otago-Southland Division of the Cancer Society of New Zealand Inc.

## Authors' contributions

JG carried out the analyses and compiled earlier drafts under supervision by AIR and RM. AIR had input into survey design, in collaboration with HSC staff; contributed to study conception and design; provided input into prior drafts and was responsible for the final submitted draft. RM contributed to the conception and all drafts. HD was responsible for an initial concept paper and contributed to each draft. All authors read and approved the final manuscript.

## Authors' information

JG (MPH) is employed at the CDC in the People's Republic of China. AIR (PhD) is Director of the Cancer Society Social and Behavioural Research Unit. RMcG (PhD) is Professor of Health Promotion in the Department of Preventive & Social Medicine which hosts the SBRU. HD (PhD) is now CEO of Oritain Global Ltd. and a Director of Forensic Solutions NZ Ltd.
